# Nanostructures for sensors, electronics, energy and environment III

**DOI:** 10.3762/bjnano.8.154

**Published:** 2017-07-27

**Authors:** Nunzio Motta

**Affiliations:** 1 School of Chemistry, Physics and Mechanical Engineering and Institute for Future Environments, Queensland University of Technology, 2 George St., Brisbane 4001, Australia

**Keywords:** biosensors, electronics, energy, environment, gas sensors, solar cells

This Thematic Series on nanostructures for sensors, electronics, energy and environment is the third edition of the successful series born from the NanoS-E3 conference. The areas of nanoscale science and technology are continually evolving, leading to new approaches for the creation and characterization of nanoscale components.

Nanotechnology spans from the foundation of atomic-scale devices, such as those developed for quantum computing, where single atoms are placed at desired locations (as suggested by Feynman in his famous speech at the American Physical Society meeting in 1959 [1]), to the realm of medicine and pharmacology, where nanoparticles are the carriers of drugs targeted on the disease (i.e., only the sick cells). In the middle there is a plethora of different materials and approaches, including nanotubes and two-dimensional materials such as graphene, and graphene-like materials (e.g., silicene, phosphorene, transition metal dichalcogenides, MXenes), which now number more than 6,000.

The topic of nanoparticles is the focus of this Thematic Series, the use of which spans from biosensing to gas detection and from removing pollutants from water to new generations of solar cells.

The interaction between light and plasma electrons generated by gold nanoparticles is critical for the development of biosensing platforms [2] and for sensors based on surface enhanced Raman scattering [3]. New methods for creating thin films are expected to provide enhanced efficiency in solar cells [4] at a fraction of the cost. This is a growing research field which is very promising to help solve the increasing demand for clean energy in the modern world.

These applications are changing our world, little by little, allowing for example for smaller, less expensive and lighter sensing devices. These, together with more powerful batteries and supercapacitors connected in a smart way to the network, will be part of the “internet of things”, allowing ubiquitous environmental and health monitoring with immediate access to this critical information from anywhere in the world.

Nanotechnology is bringing forth a technological revolution, which has parallel only in the discovery of thermal engines and in the advent of electronics. The number of applications resulting from this revolution was not imaginable even ten years ago, and this Thematic Series is a small contribution to this goal.

Nuzio Motta

Brisbane, June 2017
